# Investigation of the Ballistic Performance of GFRP Laminate under 150 m/s High-Velocity Impact: Simulation and Experiment

**DOI:** 10.3390/polym13040604

**Published:** 2021-02-17

**Authors:** Fengyan Chen, Yong Peng, Xuanzhen Chen, Kui Wang, Zhixiang Liu, Chao Chen

**Affiliations:** 1 Key Laboratory of Traffic Safety on Track (Central South University) Ministry of Education, School of Traffic & Transportation Engineering, Central South University, Changsha 410075, China; fychen@csu.edu.cn (F.C.); yong_peng@csu.edu.cn (Y.P.); kui.wang@csu.edu.cn (K.W.); lzxqj2005@163.com (Z.L.); 2 Joint International Research Laboratory of Key Technology for Rail Traffic Safety, Central South University, Changsha 410075, China; 3 National & Local Joint Engineering Research Center of Safety Technology for Rail Vehicle, Central South University, Changsha 410075, China; 4 Hunan Industry Polytechnic, Changsha 410208, China; hngyzycc@163.com

**Keywords:** GFRP laminate, high-velocity impact, finite element analysis, orthogonal test

## Abstract

The ballistic resistance of GFRP laminates subjected to high-velocity impact was studied. Based on the damage situation of GFRP laminate observed from the single-stage gas gun testing, the three-dimensional (3D) model combining strain rate effect and Hashin failure criterion was established, and the result presented good agreement between the simulation and experiment. Three factors, including layer angle, stacking sequence and proportion of different layer angles, were taken into consideration in the models. An orthogonal test method was used for the analysis, which can reduce the number of simulations effectively without sacrificing the accuracy of the result. The result indicated a correlation between the ballistic resistance and layouts of GFRP laminates, on which the stacking sequence contributed stronger influence. What was more, the laminate with layer angles 0°/90° and ±45° presented greater ballistic resistance than the other angle pairs, and adopting an equal proportion of different layer angles is helpful for GFRP laminates to resist impact as well.

## 1. Introduction

Possessing high specific strength and high specific modulus in the fiber direction, and high strength-to-weight ratios compared to traditional materials [1,2], composite materials are increasingly used in the areas of aerospace [3,4], transportation and construction [5,6] and gradually becoming a well-known topic. Especially, the dynamic testing of the 600-km/h prototype maglev train developed by CRRC Qingdao Sifang Co. was completed recently. It is foreseeable that lightweight composite materials will play an increasingly important role in future rail transit vehicle manufacturing. However, foreign object impacts such as bird strikes, hailstone strikes or other high-velocity impacts during the driving process of high-speed trains and aircrafts can significantly weakening of the strength of the body structure [7,8,9]. It is necessary to investigate the dynamic and high-velocity impact response of composite materials.

Researchers have carried out many high-speed impact studies of composite materials through experiments and simulations. For the anisotropy of continuous fiber-reinforced composites, an experiment is the most effective way to investigate the mechanical properties of them [10,11]. Researchers have carried out high-velocity impact experiments and found that composites had high ballistic resistance [12]. What is more, composites exhibit different mechanical properties at different strain rates. Generally, there is a positive correlation between Young’s modulus and strain rate, while a failure strain shows the opposite relationship [13,14,15,16]. Additionally, composite materials have been found to feature multiple failure modes under high-speed impacts, mainly by a damaged fiber fracture, matrix fracture and delamination [17]. The ballistic properties of laminate are affected by many factors, such as impacting energy, shape and impact angle of the impactor and stacking sequence [18]. Mehmet et al. [19] discussed the damage modes and the damage process of laminates under varied impact energies and chose two different stacking sequences for comparison, proving that the penetration threshold has something to do with stacking sequences. Joseph et al. [20] investigated the effect of the nose shape of the penetrator on fragments penetrating GFRP and found that the ballistic limit and energy absorption were significantly affected. Sikarwar et al. [21] studied the effects of fiber orientation and thickness on the ballistic limit and energy absorption of laminates by high-velocity impact experimentation. They found that the laminate with a 0/90° layout showed a better ballistic resistance and failure strain change with different fiber directions, whereas the impact behavior of the fiber mental laminate (FML) under normal and oblique impacts were discussed by Chen et al. [22], and they found that the lowest ballistic limit was observed when the impact angel was 30°.

Compared with these experiments, the simulation shows great benefits in reducing the cost [23]. Once the finite element (FE) model is validated by experimentation, it can be used in different conditions, which can eliminate excessive experimental testing. What is more, FE models can present the process of intralaminar failure, which is barely visible from the experiments. Some researchers have carried out related studies. Ansari et al. [24,25,26] developed a three-dimensional FE model for a progressive damage analysis of GFRP composites and presented the damage evolution and propagation of the laminate under ballistic loads. The simulation results showed good agreement with the experiment. On the other hand, they discussed the energy absorption and damage pattern of GFRP laminate under different projectile nose shapes, incidence angles, aspect ratios and boundary conditions of the model validated before. Zhang et al. [27] established a nonlinear dynamic finite element model to discuss the energy absorption and damage of FMLs from oblique impacts and found that the residual velocity of the impactor and the energy absorption of fiber mental laminates were relevant to the initial velocity and impact angle of impactors. Higuchi et al. [28] put forward an experiment and phenomenological mesomodeling and compared it with two conventional models; the results showed that the mesomodel had an advantage over others in terms of computational efficiency and damage prediction. 

Although many relevant studies have been carried out, they generally suffer from the following shortcomings: First, most previous studies about the ballistic performances of GFRP laminates have been concentrated on the energy absorption mechanism [19,21,24]; few of them focus on the out-of-plane deformations with different lay-outs. For the materials used in the train body, the out-of-plane deformations caused by high-velocity impacts might squeeze parts inside and bring about more serious losses. Second, it is foreseeable that lightweight composite materials will play an increasingly important role in future rail transit vehicle manufacturing, but there are few studies focused on impact responses when using the running speed of the latest maglev train (500–600 km/h). Therefore, relevant experiments and numerical simulations are necessary to investigate the mechanical responses of GFRP laminates under high-velocity impacts. 

In this paper, the high-velocity impact response of GFRP is investigated by experiments and simulations. The experiment is carried out at an impact speed of 150 m/s with help of a single-stage air gun, and the photographs of the impact process are obtained from a high-speed camera. The finite element model is established by using ABAQUS/Explicit at the same experimental conditions and verified by the experimental results. The finite element analysis is also conducted to study the delamination distribution and maximum deflection during the impact process in the target laminate due to different layouts. To reduce the number of simulations effectively without sacrificing the accuracy of the results, an orthogonal test method was used on the simulation. The results can be used for the further design and optimization of the GFRP laminate for high-speed train applications.

## 2. Materials and Methods 

### 2.1. High-Velocity Impact Test 

#### 2.1.1. Materials 

The rectangle laminate tested in the experiment was unidirectional glass fiber reinforced polymer laminate fabricated by using glass fiber SE4805-1200tex and polypropylene resin, which was produced by Zhuzhou Feilu High-tech Materials Co., Ltd., Zhuzhou, China. The geometric size of it was 200mm×200mm×4.8mm (ply thickness *t* = 0.3 mm). The layer configuration of the GFRP laminate is ${\left[0{}^{\circ}/90{}^{\circ}\right]}_{8S}$.

The spherical projectile adopted in the experiment was made of stainless steel. The mass and diameter of it were 33 g and 20 mm, respectively. The basic mechanical property parameters of the projectile are shown in Table 1.

#### 2.1.2. Test Method 

**Figure 1:**
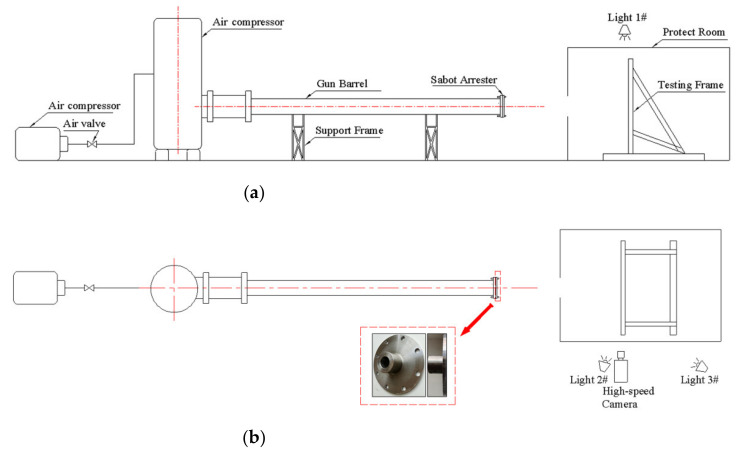
Experimental setup of the (**a**) front view and (**b**) top view.

The high-velocity impact test of GFRP laminates was carried out at Key Laboratory of Traffic Safety on Track, Ministry of Education at Central South University, Changsha, China. As shown in Figure 1, the test system consisted of a single stage gas gun and a high-speed camera. The single-stage gas gun system is mainly made up of a pressure vessel, a gun barrel, launch controls and other ancillary equipment. The pressure vessel used in the test had a volume of 0.6 $m^{3}$ and the inner pressure of it varied from 0 to 1.3 MPa. The barrel was 3 m long, with a caliber of 95 mm. The high-speed camera placed beside the protective room was shot at a frequency of 20,000 frames per second to capture the velocity of the projectile during the impacting process.

**Figure 2:**
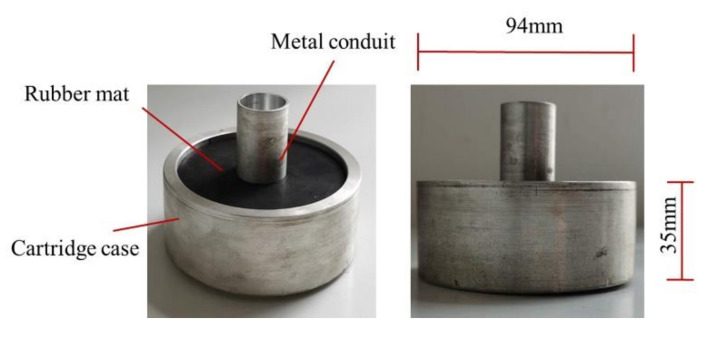
Projectile sabot.

Before the test, the projectile was preloaded into the aluminum alloy cylindrical sabot embedded with a rubber mat and metal conduit inside (Figure 2) and placed into the barrel next to the vacuum poppet valve. The laminate with the size of 200mm×200mm×4.8mm was fixed at the testing frame. Once the pressure valve was open, the released gas in the pressure vessel accelerated the sabot along the barrel to the target speed. When the sabot, together with the projectile, reached the end of the barrel, the sabot arrester at the end of the barrel forbid the sabot from moving forward, allowing the projectile to pass through the metal conduit to the composite laminate. Five repeated tests were conducted, and the average results were reported in this study.

### 2.2. Numerical Modeling

#### 2.2.1. Constitutive Model

The constitutive models used for the GFRP materials were orthotropic constitutive equations. The relationship between stress and strain is described as:

(1) $$\left[\begin{array}{c} \begin{array}{c} \varepsilon_{11}\\ \varepsilon_{22} \end{array}\\ \begin{array}{c} \varepsilon_{33}\\ \gamma_{12} \end{array}\\ \begin{array}{c} \gamma_{23}\\ \gamma_{13} \end{array} \end{array}\right]=\left[\begin{array}{cccccc} \frac{1}{E_{11}} & -\frac{\nu_{21}}{E_{22}} & -\frac{\nu_{31}}{E_{33}} & 0 & 0 & 0\\ -\frac{\nu_{12}}{E_{11}} & \frac{1}{E_{22}} & -\frac{\nu_{32}}{E_{33}} & 0 & 0 & 0\\ -\frac{\nu_{13}}{E_{11}} & -\frac{\nu_{23}}{E_{22}} & \frac{1}{E_{33}} & 0 & 0 & 0\\ 0 & 0 & 0 & \frac{1}{G_{12}} & 0 & 0\\ 0 & 0 & 0 & 0 & \frac{1}{G_{23}} & 0\\ 0 & 0 & 0 & 0 & 0 & \frac{1}{G_{13}} \end{array}\right]\left[\begin{array}{c} \begin{array}{c} \sigma_{11}\\ \sigma_{22} \end{array}\\ \begin{array}{c} \sigma_{33}\\ \tau_{12} \end{array}\\ \begin{array}{c} \tau_{23}\\ \tau_{31} \end{array} \end{array}\right]$$

where $E_{ii}$, $\nu_{ij}$ and $G_{ij}$ are Young’s modulus, Poisson’s ratios and shear modulus of the longitude and transverse direction. In the elastic stage, the stress and strain have a positive correlation. The mechanical parameters of the GFRP laminates are displayed in Table 2, where $E_{11}$,$\;E_{22}$, $X_{t}$ and $Y_{t}$ are obtained by a tensile test of the GFRP laminate, and the rest of the parameters were from Reference [29]. 

#### 2.2.2. Intralaminar Damage Model

Based on extensive research in previous works, it can be concluded that the impact damage of GFRP laminate contains intralaminar damage and interlaminar delamination. In terms of intralaminar damage, there were four types of damage failure modes produced during the impact process, including the tensile fracture of fibers, compressive fracture of fibers, tensile fracture of the matrix and compressive fracture of the matrix [30]. The damage occurred when the stress satisfied the rules for [31]:

Fiber tensile damage $\left(\sigma_{11}\geq 0\right)$:

(2) $$F_{f}^{t}={\left(\frac{\sigma_{11}}{X_{t}}\right)}^{2}+\frac{\tau_{12}^{2}+\tau_{13}^{2}}{S_{12}^{2}}\geq 1$$

Fiber compressive damage $\left(\sigma_{11}<0\right)$:

(3) $$F_{f}^{c}={\left(\frac{\sigma_{11}}{X_{c}}\right)}^{2}\geq 1$$

Matrix tensile damage $\left(\sigma_{22}+\sigma_{33}\geq 0\right)$:

(4) $$F_{m}^{t}={\left(\frac{\sigma_{22}+\sigma_{33}}{Y_{t}}\right)}^{2}+\left(\frac{\tau_{23}^{2}-\sigma_{22}\sigma_{33}}{S_{23}^{2}}\right)+\frac{\tau_{12}^{2}+\tau_{13}^{2}}{S_{12}^{2}}\geq 1$$

Matrix compressive damage $\left(\sigma_{22}+\sigma_{33}<0\right)$:

(5) $$F_{m}^{c}={\left(\frac{\sigma_{22}+\sigma_{33}}{2S_{23}}\right)}^{2}+\left[{\left(\frac{Y_{c}}{2S_{23}}\right)}^{2}-1\right]\cdot \frac{\sigma_{22}+\sigma_{33}}{Y_{c}}+\left(\frac{\tau_{23}^{2}-\sigma_{22}\sigma_{33}}{S_{23}^{2}}\right)+\frac{\tau_{12}^{2}+\tau_{13}^{2}}{S_{12}^{2}}\geq 1$$

where $X_{i}$, $Y_{i}$ and $Z_{i\;}$ $\left(i=t,\;c\right)$ are the strength in the fiber direction, vertical fiber direction and thickness direction, respectively. Subscript i denotes different statuses, i=t represents tensile and i=c represents for compressive. $S_{ij}$ (i,j=1, 2, 3) stands for the shear strength of the longitude and transverse direction.

Strain rate behavior is an important factor in high-velocity impacts. The following formula gives the relationship between the strain rate and strength of GFRP laminates [32]:

(6) $$\left\{\begin{array}{c} S=S_{0}\left(1+C_{1}{\dot{\varepsilon }}^{C_{2}}\right)\;\dot{\varepsilon }<300s^{-1}\\ S=S_{0}\left(1+C_{3}ln\frac{\dot{\varepsilon }}{\dot{\varepsilon_{0}}}\right)\;\dot{\varepsilon }\geq 300s^{-1} \end{array}\right.$$

where S stands for the current strain rate strength, and $S_{0}$ is the strength at the corresponding reference strain rate. $C_{i}$ (i=1, 2, 3) represents the strain rate hardening correction coefficient. The values used in the paper were 0.74, 0.13 and 0.2, respectively [32].

**Figure 3:**
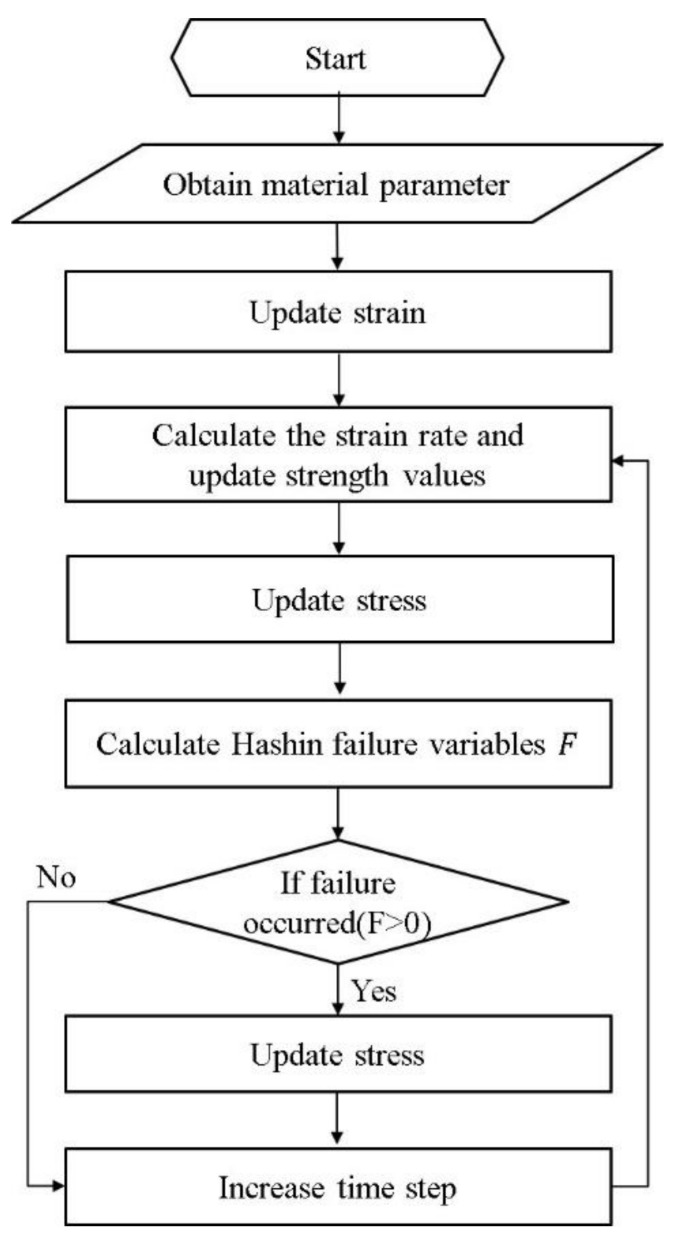
Flowchart of VUMAT for calculating the impact response of the GFRP laminate.

ABAQUS/Explicit provides users with a subroutine that can be used to customize the constitutive materials and failure models. A user-defined material subroutine (VUMAT) was compiled in the present study. This procedure is shown in Figure 3.

#### 2.2.3. Interlaminar Damage Model

A cohesive zone model (CZM) was used to simulate the interlaminar damage between layers. The behavior of the cohesive elements followed the traction–separation law. The damage occurred when the elements satisfied the following equation:

(7) $${\left\{\frac{\tau_{n}}{N}\right\}}^{2}+{\left\{\frac{\tau_{s}}{S}\right\}}^{2}+{\left\{\frac{\tau_{t}}{S}\right\}}^{2}=1$$

where $\tau_{n}$ stands for normal stress, $\tau_{s}\;\mathrm{and}\;\tau_{t}$ stand for shear stress in different directions, respectively. N and S present the normal and shear strength, respectively. The damage evolution of the elements follows the Benzeggagh-Kenane (B-K) fracture criterion, which is given as:

(8) $$\Gamma^{C}=\Gamma_{n}^{C}+\left(\Gamma_{s}^{C}-\Gamma_{n}^{C}\right){\left(\frac{\Gamma_{s}+\Gamma_{t}}{\Gamma_{n}+\Gamma_{s}+\Gamma_{t}}\right)}^{\eta }$$

where $\Gamma_{n}^{C}$ and $\Gamma_{s}^{C}$ are the critical strain energy release rates in failure modes I and II. $\Gamma_{n}$, $\Gamma_{s}$ and $\Gamma_{t}$ are fracture energy along the normal, shear and transverse directions. η is the material parameter of the B-K fracture criterion. The mechanical properties of the cohesive elements are shown in Table 3 [33].

#### 2.2.4. Numerical Model 

**Figure 4:**
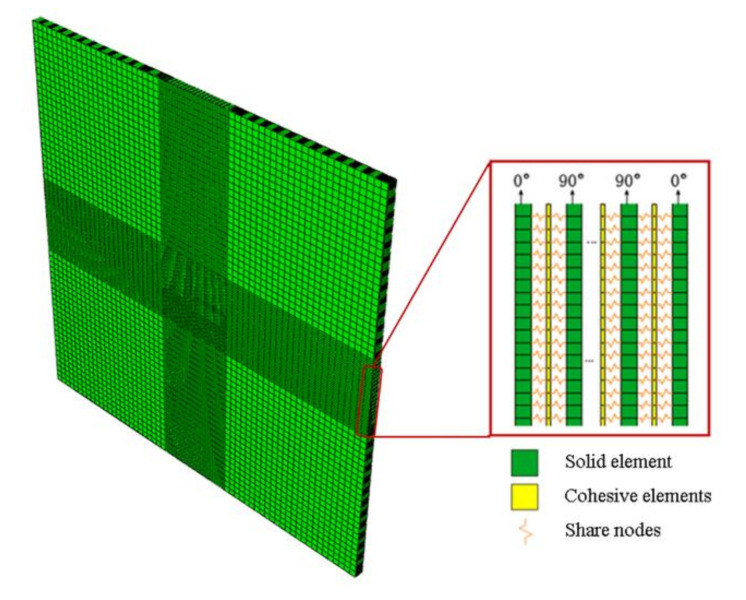
The model setup in the simulation.

The high-velocity impact process of GFRP laminate was simulated using finite element analysis software. Two parts were created in the model, including the solid elements of the laminate and projectile. The interlaminar delamination must be taken into consideration between two different layouts of the laminate. Hence the cohesive elements were inserted into different layouts layers with the same geometric sizes of the GFRP layers. Their failure evolutions followed the rules shown in Equations (7) and (8). The size of each part was consistent with the actual size in the experiment. The geometric size of the laminate was 200mm×200mm×4.8mm, including 16 layers (ply thickness *t* = 0.3 mm), which is shown in Figure 4. 

It was shown in the experiment that the damaged areas were mainly distributed around the impact point, so the refined mesh in the central area could improve the computational accuracy effectively. Based on the research of Zhikharev et al. [34,35], the convergence of the model obtained an optimum when the element size of the impact center was 1 mm. Therefore, the mesh size of the laminate center (40 mm × 40 mm) was set as the C3D8R element with a size of 1 mm × 1 mm, and there were 40 elements along the length and width of the laminate. The remaining part of the laminate was discrete, with 4-mm elements. As no obvious deformation was observed during the impact, the spherical projectile was modeled as a rigid body. The shape and weight of the projectile in the model were the same as the experiment. The boundary conditions used in the model were that the laminate edge nodes were fixed, and all degrees of freedom were set to zero. On the basis of the experiment data, the initial velocity of the projectile was set to 150 m/s. To simulate the interaction between the projectile and GFRP laminate, general contact was used with a friction coefficient of 0.3.

## 3. Results 

### 3.1. High-Velocity Impact Test Results 

**Figure 5:**
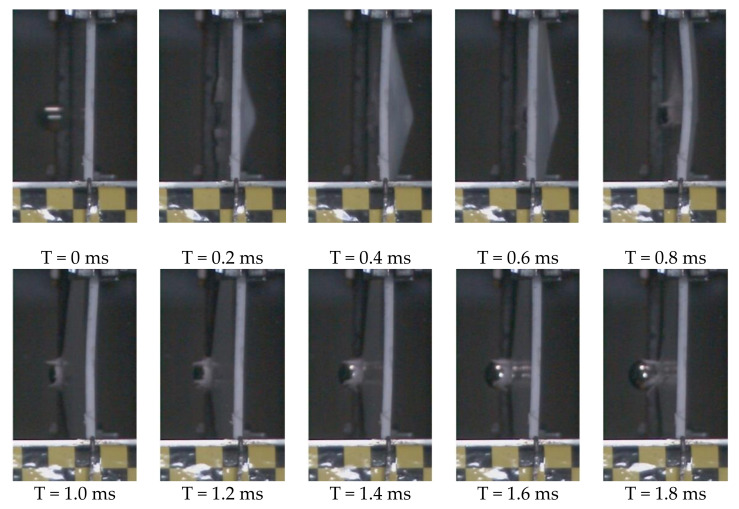
The process of GFRP laminate under the impact of a steel projectile captured by a high-speed camera.

**Figure 6:**
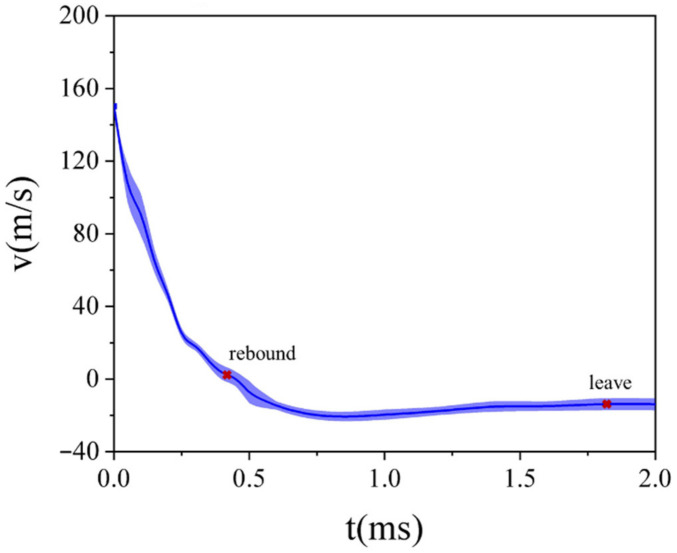
The velocity–time curve (with error band) of the projectile impacting the GFRP laminate at the velocity of 150 m/s.

The impact process of GFRP laminate subjected to high-velocity impact of the projectile recorded by a high-speed camera is shown in Figure 5. The projectile contacting the laminate at 0 ms and the impact process lasted for approximately 1.8 ms. With the aid of the analysis software matched with the high-speed camera, the velocity of the projectile was recorded, and the velocity–time curve (with error band) is plotted in Figure 6. The impact process can be divided into two stages: At first, the velocity of the projectile drops sharply when it contacts the GFRP laminate and the laminate bulge at the impact point. Then, the deflection of the GFRP laminate keeps increasing as the projectile moves forward, until the velocity of the projectile is reduced to zero at 0.45 ms. In the second stage, the projectile starts to rebound. The velocity of it increases slowly. Finally, at 1.8 ms, the projectile leaves the laminate at a speed of 13.88 m/s. 

**Figure 7:**
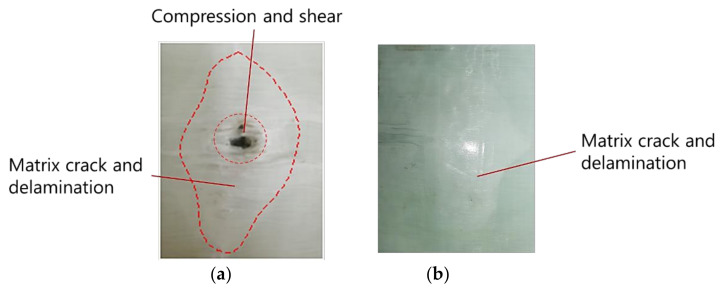
Visible damage morphologies of the (**a**) front surface and (**b**) rear surface after the projectile impact.

The tested GFRP laminate was inspected visually, as shown in Figure 7. The results showed that the laminate was not penetrated when subjected to the impact velocity of 150 m/s, and the damage area was mainly concentrated on the impact point. From Figure 7, the damage zone of the front layers can be divided into two areas: the first area is marked by a smaller circle in Figure 7a, including the compression failure below the projectile and fiber breakage around the edge of the compression zone. When the projectile contacted the target, the laminate generated normal stress in the thickness direction and shear stress on the edges of the contact face in order to resist deformation, which caused the damage above. The other damage zone of the front layers was a matrix crack and delamination, which can also be seen on the rear layers. What was more, the delamination between the rear layers was greater than the front ones, and we will discuss that later.

### 3.2. Numerical Results and Comparisons with the Experiments

**Figure 8:**
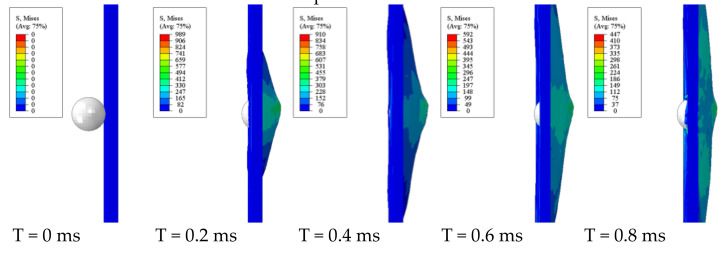

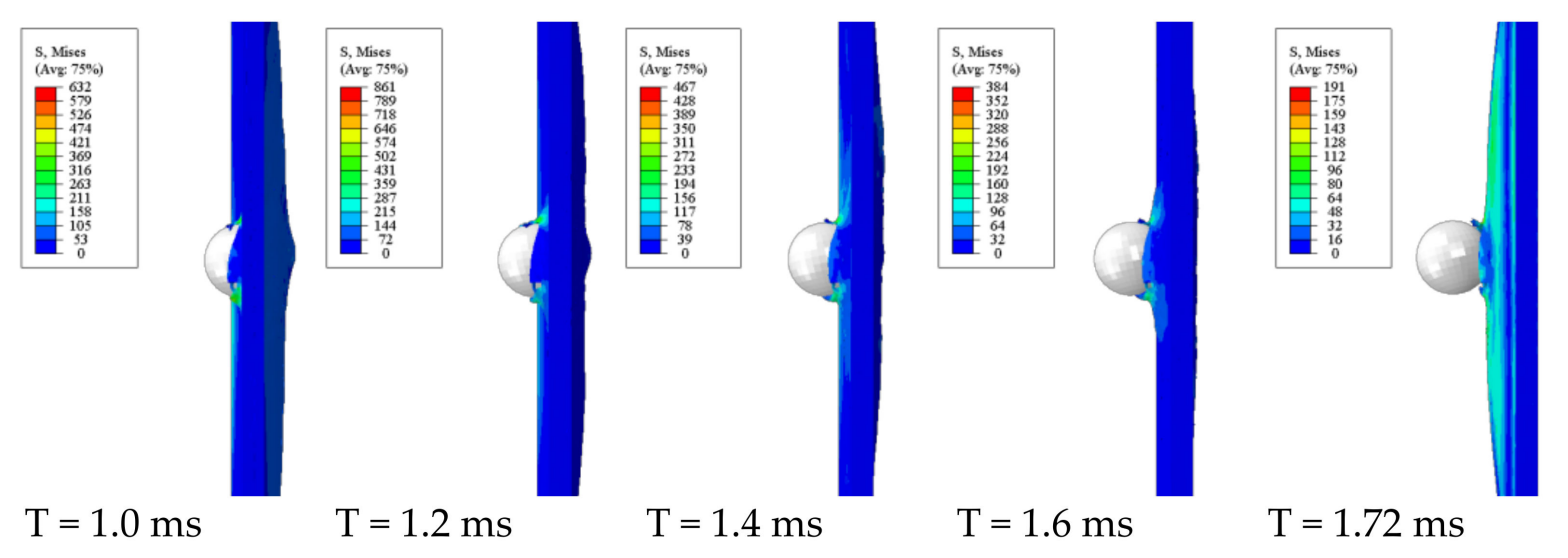
The physical process of the steel projectile impacting the GFRP laminate at a velocity of 150 m/s.

Figure 8 displays the simulation results of the physical process of the steel projectile impacting the GFRP laminate at a velocity of 150 m/s. The impact process in the simulation lasted for 1.72 ms, which was almost same as the experiment. At first, the laminate bulged at the impact point; as the projectile moved forward, the failure elements were generated, contributing to the break in the front layers. After that, the delamination happened gradually. At 0.4 ms, the projectile rebounded, and it finally left the laminate at 1.72 ms with a speed of 14.84 m/s.

**Figure 9:**
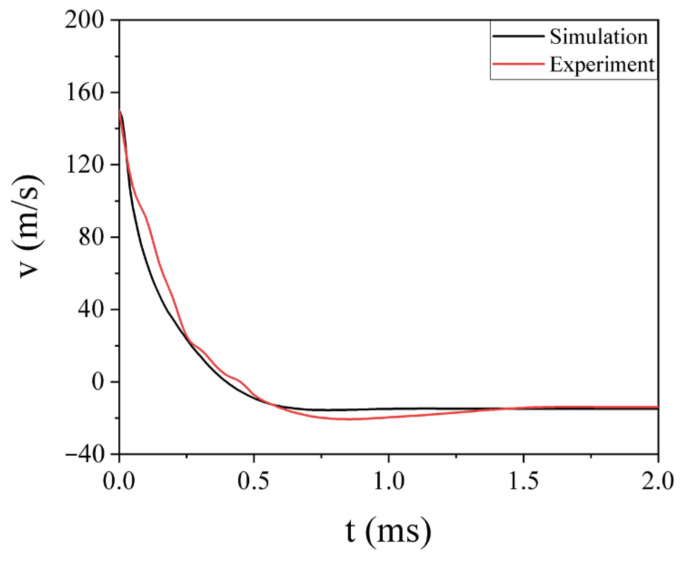
Comparison between the simulation and experiment of the velocity of the projectile.

**Figure 10:**
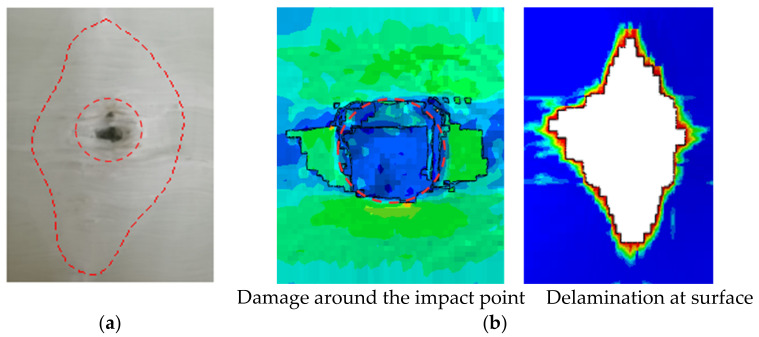
Comparison between the (**a**) experiment and (**b**) simulation of the damage distribution.

The velocity–time curve of the whole process of the projectile was compared with the one from the experiment in Figure 9. It showed a good agreement between the experiment and simulation: the trends were consistent, and the residual velocity of them was close, with an error of 6.9%. The comparison of the damage distribution between the simulation and experiment is shown in Figure 10. In the simulation, the element failure deletion occurred in the center of the laminate within the first few layers; the damage shape of the simulation was similar to the experiment results. The delamination at the surface had a rhombic appearance, which was consistent with the experiment as well. Based on the above comparison, the model accurately reproduced the impact process of the laminate, so it can be used for the research of impact resistance.

## 4. Discussion 

### 4.1. Delamination 

**Figure 11:**
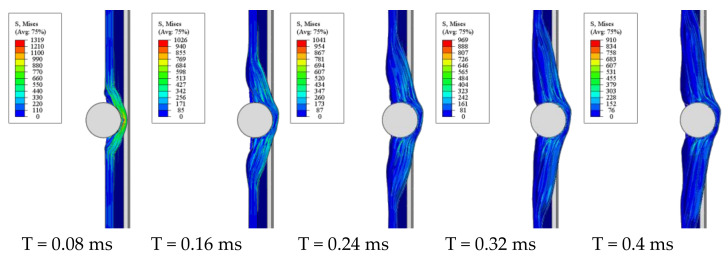
The process of delamination occurring and spreading in the GFRP laminate at 0–0.4ms.

After the projectile rebounds, the delamination of the laminate does not continue to expand, which means that the rebound process hardly affects the delamination progress. Therefore, the occurrence and expansion of the delamination are studied in the impact process during 0–0.4 ms. The process is shown in Figure 11. At first, the delamination only occurred in the first two layers; as the projectile moved forward, the deflection of the GFRP kept increasing, and the delamination spread to the back layers gradually. Most of the projectile inserted into the laminate at 0.4 ms. The delamination between the rear layers was greater than the front ones, which was due to the difference in damage type and deformation between them. Generally, the damage mode of the front layers was mainly compressive damage, while the damage type of the rear layers was mainly tensile damage. It is thought that the bending tensile stress wave keeps attenuating when it propagates from the rear layers to the front layers. On the other hand, the deformation of the rear layers was greater than the front layers, which might aggravate the separation of the layers as well.

### 4.2. Ballistic Resistance of GFRP Laminates with Varying Layouts

The effects of varying layouts (including the layer angle, stacking sequence and proportion of layer angles) on the impact response of the GFRP laminate was explored using the model established above.

In order to reduce the number of simulations effectively without sacrificing the accuracy of the result, an orthogonal test method was adopted for the analysis. The orthogonal test method is suitable for multifactor and multilevel research. On the basis of orthogonality, a series of presentable points were selected from the comprehensive test, and they were required to keep uniformly dispersed and neatly comparable in the orthogonal test [36]. Generally, an orthogonal table is used in the orthogonal test method, and the selection of it is based on the factors and levels in the test. In this work, three levels were selected for the stacking sequence, layer angle and proportions of the different layer angle pairs, respectively. The factors and levels of the orthogonal test are listed in Table 4, and the orthogonal test data is listed in Table 5.

**Figure 12:**
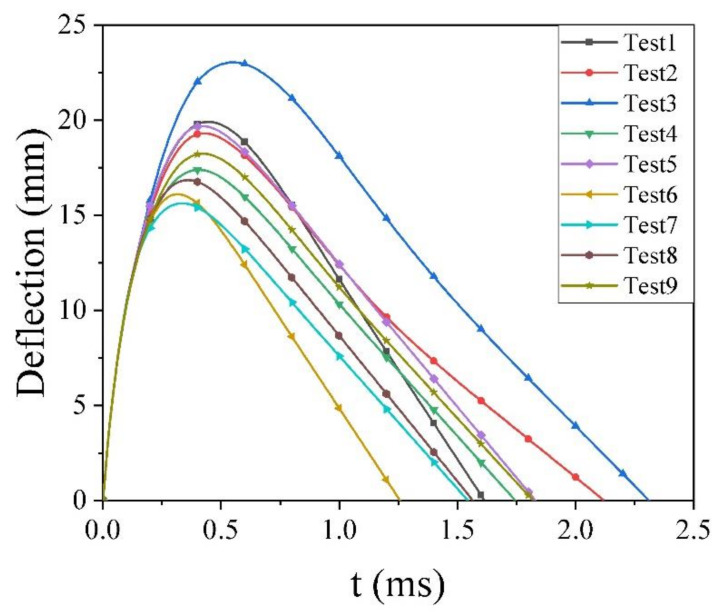
The deflection of GFRP laminates with varying layouts under the impact speed of 150 m/s.

Figure 12 shows the deflection–time curves of GFRP laminates with varying layouts. At the impact speed of 150 m/s, all the GFRP laminates in the orthogonal test are not penetrated. The deflection versus time curve of the laminate with varying layouts was kept the same trend and the max deflection fluctuate in the range of 15 to 25 mm. The smaller deflection stands for the smaller deformation of the laminate during the impact process, which means a better ballistic resistance of the GFRP laminates. It can be inferred that there is a relationship between the ballistic resistance of the GFRP laminates and layouts. Therefore, the maximum deflection was chosen as the result for the orthogonal test, as shown in Table 5. 

**Figure 13:**
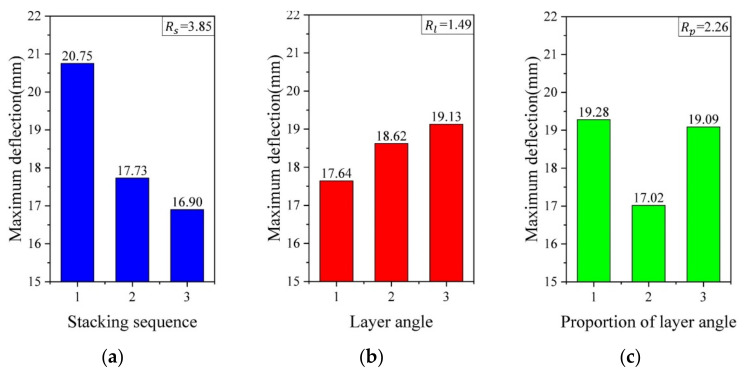
The average value of per level for the (**a**) stacking sequence, (**b**) layer angle and (**c**) proportion of the layer angle.

To explore the influence degree of three factors on the simulation results, a range analysis method was adopted in the present study. The average value $\bar{T_{n}^{x}}$ and range $R_{x}$ in the range analysis method are given as:

(9) $$\bar{T_{n}^{x}}=\;\frac{1}{m}\;\overset{k}{\underset{i=1}{\sum }}y_{n}$$

(10) $$R_{x}=max\left\{\bar{T_{n}^{x}}\right\}-min\left\{\bar{T_{n}^{x}}\right\}$$

where y is the exact value of the maximum deflection in the simulation; Subscript *x* (*x=s, l, p*) stands for the *t* factor and *s, l* and *p* represent the stacking sequence, layer angles and proportions of the layer angle, respectively. *n* is the level of each factor, and *m* represents the number of repetitions of each factor at each level. According to the analysis method above, the range analysis for the orthogonal test results is shown in Figure 13.

Comparing the range values under different cases, it can be concluded that the stacking sequence contributes a stronger influence on the maximum deflection of the GFRP laminates during the impact process; the proportion of the layer angle is the secondary factor, while the layer angle is the last one. The best combination scheme of the simulation results is ${\left[0/45/90/-45\right]}_{2s}$, and the maximum deformation of it reduced laminate 3 by 32.16%.

As shown in Figure 13a, in terms of stacking sequence, the deformation of the stacking sequence $X_{1}Y_{1}X_{2}Y_{2}$ was 16.90 mm, which decreased the stacking sequence $X_{1}X_{2}Y_{1}Y_{2}\;\mathrm{by}\;18.55\%;$ therefore, making two pairs of layer angles stack crosswise can further enhance the ballistic resistance of the GFRP laminates. The stacking sequence above helps reduce the interlaminar stress during impact. In Figure 13b, 0°/90°/45°/−45° shows the minimum deflection of the laminate, and 45°/−45°/60°/−60° has the poorest one. This can be explained that 0°/90° aligned with both the length and the width of the plate, while 45°/−45° are the middle angles between them. Therefore, the layer angle above makes the performance of anisotropic GFRP laminate similar to the isotropic material macroscopically, avoiding the properties of laminate becoming weak in some direction, which causes the earlier breaking of the laminate. Therefore, the combination of 0°/90° and ±45° can improve the impact properties of the GFRP laminates. Figure 13c presents that the equal proportions of different layer angles better bring about the ballistic resistance of GFRP laminate due to the even distribution of stress.

According to Figure 13, the best combination scheme is the combination of stacking sequence $X_{1}Y_{1}X_{2}Y_{2}$, layer angle 0°/90°/45°/−45° and an equal proportion of different layer angles, respectively, which was test 7 of the simulation. The simulation results showed that test 7 had the minimal deformation of the GFRP laminates during the impact process. Therefore, the analysis results of the orthogonal experimental test are considered as reliable.

**Figure 14:**
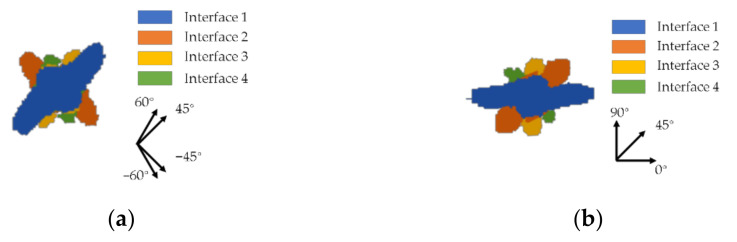
The delamination occurred in the front layers of the GFRP laminates with layout (**a**) test 3 and (**b**) test 7 at 0.05 ms.

**Figure 15:**
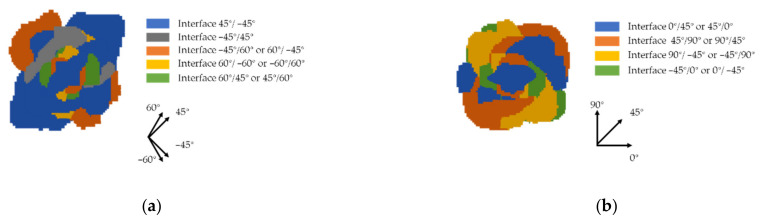
The delamination distribution of (**a**) test 3 and (**b**) test 7 after impact.

Based on the research of Carraro [37] and Sztefek [38], delamination will bring about the degradation of the laminate stiffness, and the stiffness gradually decreases towards the damage area; this can be used to explain the difference in the maximum deflection of the GFRP laminate with different layouts. The delamination distribution of test 3 (with maximum deflection) and test 7 (with minimal deflection) are shown in Figure 14 and Figure 15. Figure 14 shows the delamination occurring in the front layers at 0.05 ms. It can be obtained that the delamination expands along the fiber direction of the adjacent layers. The damage distribution of laminate 7 is disperse, while, in laminate 3, there is a larger overlapping area of the delamination damage of the nearby interface. The overlap area stands for the accumulation of damage, where the laminate stiffness will be severely degraded. Therefore, laminate 3 presented a higher deflection, where the expansion of delamination followed the direction of the fibers in adjacent layers. That means that, when the interface angle is small, the overlapping area of the delamination damage will increase, and the ballistic performance of the material in this area will become weaker. Figure 15 presents the damage distribution of the GFRP laminates after impact. The delamination distribution of laminate 7 is disperse, and the total delamination distribution is circular, which also proves the quasi-isotope of the laminate. In test 3, the total delamination distribution is shown to be “X”-shaped, which is also the weaker area in the laminate. What is more, the delamination is easy to expand in that area, which indicates that the degradation of the stiffness will also aggravate the expansion of the delamination damage of the laminate. 

## 5. Conclusions

In this study, the high-velocity impact research of unidirectional GFRP laminates under the impact velocity of the running speed of the latest maglev train (500–600 km/h) was carried out in an experiment and a finite simulation in ABAQUS. The primary conclusions drawn are as follows:

● Determination of the material model: Through the analysis of the experiment results, the finite element model combining the strain rate effect and Hashin failure criterion was determined.
● Impact response of the GFRP laminate and numerical simulations based on the FE: The FE simulation was established by the material model selected in the paper. The results presented good agreement between the simulation and experiment, which confirmed that the selected material model was reasonable.
● Analysis of the intralaminar and interlaminar damages: (1) Different types of failures were produced for the high-velocity impact test, including the compression damage below the projectile and fiber breakage on the periphery of the projectile caused by normal stress and shear stress, respectively. The delamination happened in both the front and rear layers. (2) The expansion of delamination mainly occurred in the penetration stage, and the delamination between the rear layers was greater than the front ones, which was due to the difference in damage type and deformation between them.
● Analysis of the deformation: (1) For laminates with different layouts, it can be concluded that the stacking sequence contributes a stronger influence on the maximum deflection of the GFRP laminates. The proportions of the layer angle are the second-most important factor, while the layer angle is the least one. The best combination scheme was inferred as ${\left[0/45/90/-45\right]}_{2s}$, and the maximum deformation of it reduced laminate 3 by 32.16%. (2) The larger overlapping area of the delamination damage resulted in a higher deflection, so there was no recommendation for the laminate with a too-small interface angle. The quasi-isotope showed a better ballistic performance.

## Figures and Tables

**Table 1:** The mechanical properties of the projectile.

Material	Diameter (mm)	Destiny (kg/$m^{3}$)	Mass (g)	Elastic Modulus (GPa)	Poisson’s Ratio
Stainless Steel	20	7980	33	206	0.30

**Table 2:** The mechanical properties of GFRP laminate.

Parameter	$E_{11}\;\left(\mathrm{MPa}\right)$	$E_{22}\;\left(\mathrm{MPa}\right)$	$\nu_{12}$	$G_{12}\;\left(\mathrm{MPa}\right)$	$G_{23}\;\left(\mathrm{MPa}\right)$
Value	28,000	3400	0.064	946	1598
Parameter	$X_{t}\;\left(\mathrm{MPa}\right)$	$X_{c}\;\left(\mathrm{MPa}\right)$	$Y_{t}\;\left(\mathrm{MPa}\right)$	$Y_{c}\;\left(\mathrm{MPa}\right)$	$S_{12}\;\left(\mathrm{MPa}\right)$
Value	746	160	15	50	16

**Table 3:** The mechanical properties of the cohesive interface.

Parameter	$N\;\left(\mathrm{MPa}\right)$	$S\;\left(\mathrm{MPa}\right)$	$\Gamma_{n}^{C}\;\left(N/\mathrm{mm}\right)$	$\Gamma_{s}^{C}\;\left(N/\mathrm{mm}\right)$	η
Value	15	16	2.08	1.44	1.45

**Table 4:** Factors and levels of the orthogonal test.

Factors	Stacking Sequence	Layer Angles	Proportions of Different Layer Angle Pairs
Level 1	$X_{1}X_{2}Y_{1}Y_{2}$	AB	1:3
Level 2	$X_{1}Y_{1}Y_{2}X_{2}$	AC	1:1
Level 3	$X_{1}Y_{1}X_{2}Y_{2}$	BC	3:1

Where A stands for the angle pair 0°/90°, B stands for the angle pair 45°/−45°, and C stands for the angle pair 60°/−60°. $X_{i\;}$ and $Y_{i}$ stand for the exact angle of each pair. Subscript i denotes the relative order of the angles. *i* = 1 means the former one, and *i* = 2 means the latter. For angle pair A, *i* = 1 means 0°, and *i* = 2 means 90°. For example, the combination of $X_{1}X_{2}Y_{1}Y_{2}$–AB–1:3 stands for layout ${\left[0/90/{\left[45/-45\right]}_{3}\right]}_{s}$ of the CFRP laminate in the orthogonal test. The layout sequences of the GFRP laminates simulated in the paper are listed in Table 5.

**Table 5:** Orthogonal experimental design and test results.

No.	Stacking Sequence	Layer Angles	Proportions of Layer Angle	Layout Sequence	Maximum Deflection
1	1	1	1	${\left[0/90/{\left[45/-45\right]}_{3}\right]}_{s}$	19.92
2	1	2	2	${\left[0/90/60/-60\right]}_{2s}$	19.31
3	1	3	3	${\left[45/-45/60/-60/{\left[45/-45\right]}_{2}\right]}_{s}$	23.04
4	2	1	3	${\left[0/45/-45/90/{\left[0/90\right]}_{2}\right]}_{s}$	17.39
5	2	2	1	${\left[0/60/-60/90/{\left[60/-60\right]}_{2}\right]}_{s}$	19.69
6	2	3	2	${\left[45/60/-60/-45\right]}_{2s}$	16.11
7	3	1	2	${\left[0/45/90/-45\right]}_{2s}$	15.63
8	3	2	3	${\left[0/60/90/-60/{\left[0/90\right]}_{2}\right]}_{s}$	16.85
9	3	3	1	${\left[45/60/-45/-60/{\left[60/-60\right]}_{2}\right]}_{s}$	18.24

## Data Availability

The data presented in this study are available on request from the corresponding author.
